# Diurnal Profiles of *N*-Acylethanolamines in Goldfish Brain and Gastrointestinal Tract: Possible Role of Feeding

**DOI:** 10.3389/fnins.2019.00450

**Published:** 2019-05-07

**Authors:** Miguel Gómez-Boronat, Esther Isorna, Andrea Armirotti, María J. Delgado, Daniele Piomelli, Nuria de Pedro

**Affiliations:** ^1^Departamento de Genética, Fisiología y Microbiología, Unidad Docente de Fisiología Animal, Facultad de Biología, Universidad Complutense de Madrid, Madrid, Spain; ^2^Analytical Chemistry Laboratory, Istituto Italiano di Tecnologia, Genoa, Italy; ^3^Departments of Anatomy and Neurobiology, Pharmacology, and Biological Chemistry, University of California, Irvine, Irvine, CA, United States

**Keywords:** OEA, PEA, SEA, acylethanolamides, PPARα, food intake, rhythms, fish

## Abstract

*N*-acylethanolamines (NAEs) are a family of endogenous lipid signaling molecules that are involved in regulation of energy homeostasis in vertebrates with a putative role on circadian system. The aim of this work was to study the existence of daily fluctuations in components of NAEs system and their possible dependence on food intake. Specifically, we analyzed the content of oleoylethanolamide (OEA), palmitoylethanolamide (PEA), stearoylethanolamide (SEA), their precursors (NAPEs), as well as the expression of *nape-pld* (NAEs synthesis enzyme), *faah* (NAEs degradation enzyme), and *pparα* (NAEs receptor) in gastrointestinal and brain tissues of goldfish (*Carassius auratus*) throughout a 24-h cycle. Daily profiles of *bmal1a* and *rev*-erbα expression in gastrointestinal tissues were also quantified because these clock genes are also involved in lipid metabolism, are PPAR-targets in mammals, and could be a link between NAEs and circadian system in fish. Gastrointestinal levels of NAEs exhibited daily fluctuations, with a pronounced and rapid postprandial increase, the increment being likely caused by food intake as it is not present in fasted animals. Such periprandial differences were not found in brain, supporting that NAEs mobilization occurs in a tissue-specific manner and suggesting that these three NAEs could be acting as peripheral satiety signals. The abundance of *pparα* mRNA displayed a daily rhythm in the intestine and the liver, suggesting a possible rhythmicity in the NAEs functionality. The increment of *pparα* expression during the rest phase can be related with its role stimulating lipid catabolism to obtain energy during the fasting state of the animals. In addition, the clock genes *bmal1a* and *rev*-erbα also showed daily rhythms, with a *bmal1a* increment after feeding, supporting its role as a lipogenic factor. In summary, our data show the existence of all components of NAEs system in fish (OEA, PEA, SEA, precursors, synthesis and degradation enzymes, and the receptor PPARα), supporting the involvement of NAEs as peripheral satiety signals.

## Introduction

Acylethanolamides or *N*-acylethanolamines (NAEs) are a family of endogenous bioactive lipid molecules present in animal, plant, as well as in prokaryotic cells (Hansen and Vana, 2018), which play a key role in feeding regulation in vertebrates (Borrelli and Izzo, 2009; Hansen, 2014; Kleberg et al., 2014; Romano et al., 2015). They consist on a fatty acid linked by an amide bound to an ethanolamine and are classified based on the number of carbons and degree of saturation of their acyl chain. Major NAEs in mammalian tissues comprise oleoylethanolamide (N-oleoylethanolamine, OEA), palmitoylethanolamide (*N*-palmitoylethanolamine, PEA) and stearoylethanolamide (N-stearoylethanolamine, SEA), and several other quantitative minor species including anandamide (*N*-arachidonoylethanolamine, AEA; Tsuboi et al., 2013).

Endogenous levels of NAEs are mainly regulated by enzymes responsible for their formation and degradation. Biosynthesis of NAEs is an “on demand” process with two major steps, the formation of *N*-acylphosphatidylethanolamines (NAPEs) from their phospholipid precursors through a Ca^2+^-dependent N-acyltransferase (NAT) activity and the conversion of NAPEs to NAEs via several pathways (Tsuboi et al., 2013; Rahman et al., 2014). In animals, most NAEs result from a NAPE hydrolysis catalyzed in a single enzymatic step by a specific membrane-bound phospholipase D, namely NAPE-PLD, although other multi-step pathways have been described (Borrelli and Izzo, 2009; Tsuboi et al., 2013; Ueda et al., 2013; Inoue et al., 2017). The generated NAEs are rapidly catabolized by fatty acid amide hydrolase (FAAH) to their corresponding free fatty acids and ethanolamine. FAAH is localized in endoplasmic reticulum and functions as a general inactivating enzyme for all NAEs in mammals, with highest activity in liver, small intestine and brain (Ueda et al., 2013; Kleberg et al., 2014; Hansen and Vana, 2018). Moreover, a NAE-hydrolyzing acid amidase (NAAA) localized in lysosomes contributes to NAEs catabolism, and preferentially hydrolyzes PEA over the other NAEs (Ueda et al., 2013).

These NAEs can interact with different receptors which are involved in many physiological processes, playing an important role in the regulation of energy homeostasis (Borrelli and Izzo, 2009; Hansen, 2014; Kleberg et al., 2014; Romano et al., 2015). The most studied NAE in mammals is OEA, which acts as an anorexigenic signal and promotes fat catabolism (Bowen et al., 2017; Sihag and Jones, 2018). In goldfish, OEA also reduces food intake and body weight, and is involved in lipid and glucose metabolism (Tinoco et al., 2014; Gómez-Boronat et al., 2016). A food intake decrease was also observed after exogenous administration of PEA or SEA in the only vertebrates studied to date, rats and mice (Rodríguez de Fonseca et al., 2001; Terrazzino et al., 2004). These functions seem to be mediated via activation of the transcription factor peroxisome proliferator-activated receptor alpha (PPARα), although OEA and PEA also bind other receptors, such as G protein-coupled receptor GPR119 and a transient receptor potential vanilloid type 1 (TRPV1; Hansen, 2010; Kleberg et al., 2014).

Fed and fasted states modulate NAEs production in vertebrates. Intestinal levels of OEA are decreased by food deprivation and increased upon refeeding in rodents (Rodríguez de Fonseca et al., 2001; Piomelli, 2013; Bowen et al., 2017) and goldfish (Tinoco et al., 2014). A similar feeding-induced PEA mobilization in small intestine, without modifications in SEA, has also been described in rats (Petersen et al., 2006); although another study reported that food deprivation does not change the duodenal or jejunal content of both PEA and SEA in this species (Fu et al., 2007). A postprandial OEA, PEA, and SEA increment has also been described in small intestine of Burmese python (*Python molurus*), the only reptile species so far studied (Astarita et al., 2006). These postprandial variations seem to occur in a tissue-specific manner, since changes in OEA and PEA levels in response to feeding were not observed in other peripheral tissues and brain structures in rats (Fu et al., 2007; Izzo et al., 2010).

Because feeding is usually a rhythmic behavior, the existence of daily fluctuations in some feeding regulators has been reported (Bechtold and Loudon, 2013; Isorna et al., 2017). However, only few studies have investigated the daily rhythmicity in the NAEs system and its possible interaction with the circadian system, with no consistent results. Diurnal fluctuations of endogenous levels of NAEs have been found in brain: while in the cerebrospinal fluid, OEA and PEA concentrations increased during the light-on period; in pons, hippocampus and hypothalamus, these NAEs increased during the dark phase in rat (Murillo-Rodriguez et al., 2006). However, OEA levels in various brain regions of mice did not change between 11:30 a.m. and 11:30 p.m. (Guijarro et al., 2010). In gastrointestinal tissues, OEA levels display diurnal fluctuations in rodents, being higher during the daytime, when animals are satiated, and lower during the night, when they are awaked and actively feeding (Fu et al., 2003; LoVerme et al., 2005; Guijarro et al., 2010). No differences were found in other components of NAEs system, such as PEA, NAPEs and activity of the enzymes NAPE-PLD and FAAH at the midpoint of the light and dark phase in mice jejunum (Guijarro et al., 2010). FAAH activity was also similar in various brain regions, with a decline only in cerebellum (Glaser and Kaczocha, 2009), striatum, and hippocampus (Valenti et al., 2004) at midnight. Although FAAH K_m_ and V_max_ are affected by time of the day in some brain regions of mice, none of the data support a primary role for FAAH in the circadian regulation of the brain NAEs (Liedhegner et al., 2014). To date, the clearest link between NAEs and the circadian system is the fact that their main receptor, PPARα, directly regulates the transcription of BMAL1 and REV-ERBα, two core clock genes, which possess a PPAR response element (PPRE) in their promoters (Charoensuksai and Xu, 2010; Chen and Yang, 2014). In addition, PPARα is also a direct target gene of the heterodimer CLOCK/BMAL1, a key component of the molecular clock which drive rhythms in target genes known as clock-controlled genes. Thus, PPARα is considered an output gene and shows daily rhythmic expression in a variety of tissues in mammals (Yang et al., 2006; Chen et al., 2010). This interaction between OEA and the circadian system has also been suggested in fish, since hepatic expression of *bmal1a* increases after OEA treatment in goldfish (Gómez-Boronat et al., 2016) and the expression of *pparα* is rhythmic in gilthead sea bream (*Sparus auratus*; Paredes et al., 2014) and zebrafish (*Danio rerio*; Paredes et al., 2015). Apart from these data, there is no evidence in fish on the possible daily rhythmicity in OEA and other NAEs.

The aim of this work was to study the existence of daily fluctuations in the NAEs system components and their possible regulation by food intake in fish. Specifically, we quantified the content of OEA, PEA, SEA, and their precursors (NAPEs) in central and peripheral tissues of goldfish throughout a 24-h cycle. The daily pattern of expression in gastrointestinal tissues of *nape-pld* (NAEs synthesis enzyme), *faah* (NAEs degradation enzyme), and *pparα* (NAEs receptor) was also measured. Moreover, the enzymatic activity of FAAH was quantified in anterior intestine and hypothalamus. Finally, the daily rhythmic expression of the clock genes *bmal1a* and *rev*-erbα was analyzed in gastrointestinal tissues to investigate a possible interaction between NAEs and the circadian system.

## Materials and Methods

### Animals and Housing

Goldfish with a body mass (bm) of 23 ± 6 g were obtained from a local commercial supplier (ICA, Madrid, Spain). Fish were housed in 60 l aquaria with filtered fresh water (21 ± 1°C) and continuous aeration and maintained under a 12 h light: 12 h darkness (12L:12D) photoperiod (lights on at 8 a.m., considered as *zeitgeber* time 0-ZT0). The aquaria walls were covered with opaque paper to minimize external interferences during the experiment. Fish were fed (1% bm) once daily at 10 a.m. (ZT2) with commercial dry pellets (32.1% crude protein, 5% crude fat, 1.9% crude fiber, 6.8% crude ash, 5.1% water, and the rest nitrogen free extract; Sera Pond, Heinsberg, Germany). Animals were maintained under these conditions for 1 month. The experiments described comply with the Guidelines of the European Union Council (UE63/2010) and the Spanish Government (RD53/2013) for the use of animals in research and were approved by the Animal Experimentation Committee of Complutense University (O.H.-UCM-25-2014) and the Community of Madrid (PROEX 107/14).

### Experimental Design

Goldfish (*n* = 49) were sampled throughout a 24-h cycle each 4 h (*n* = 7 per sampling point; ZT3, ZT7, ZT11, ZT15, ZT19, ZT23, and ZT3 of next day -ZT3b). Food was offered as scheduled (ZT2) the first day of the experiment, but not the second day before last sampling point (ZT3b). Thus, the possible effect of food intake on the NAEs system was tested by comparing fish sampled at the same time but 1-h postprandial (ZT3) or 25-h fasting (ZT3b). In each sampling point, animals were sacrificed by anesthetic overdose (tricaine methanesulfonate, MS-222, 0.28 g/l; Sigma-Aldrich, Madrid, Spain) followed by spinal cord section. Tissue were quickly dissected: initial and final segments of intestinal bulb, anterior intestine in two sections, liver in three aliquots, and central tissues (hypothalamus and telencephalon) as a whole. All samples were rapidly frozen in liquid nitrogen and immediately stored at -80°C until analysis.

### Determination of Tissue Content of NAEs and NAPEs

A longitudinal half of the final segment of the intestinal bulb, a transversal half of the initial segment of the anterior intestine, and one liver aliquot were weighed (20–30 mg) as well as a longitudinal half of both hypothalamus and telencephalon (5–10 mg). Samples were homogenized in 1 ml of methanol (Thermo Fisher Scientific, Milan, Italy) containing the following deuterated internal standards (IS): OEA–d_4_ (100 nM), PEA–d_4_ (100 nM), SEA–d_3_ (100 nM), and C17:0 NAPE (25 nM) (Cayman Chemical, Ann Arbor, MI, United States). Then, this solution was mixed with 2 *v* of chloroform (Thermo Fisher Scientific) and 1 *v* of water. Organic phase was collected, dried under nitrogen atmosphere, and fractioned by open-bed silica gel column chromatography, as previously described (Cadas et al., 1997; Tinoco et al., 2014). Briefly, the lipid extracts were reconstituted in chloroform and loaded onto small columns packed with Silica Gel G (60 Å 230–400 Mesh ASTM; Whatman, Clifton, NJ, United States). NAEs and NAPEs were eluted with a methanol:chloroform solution (1:9 and 1:1, respectively). Both eluates were again dried under nitrogen atmosphere and, subsequently, NAEs were reconstituted in 75 μl and NAPEs in 100 μl of methanol:chloroform (9:1). Samples were then analyzed by UPLC–MS/MS on a Xevo–TQ triple quadruple mass spectrometer coupled with an UPLC (ultra-performance liquid chromatography) system (Waters Inc., Milford, PA, United States). NAEs and its deuterated analogs were loaded on a reversed phase BEH C18 column (50 × 2.1 mm inner diameter, 1.7 μm particle size, maintained at 45°C; Waters Inc.) operated at a constant flow rate of 0.5 ml/min. The mobile phase consisted of 0.1% formic acid in water as solvent A and 0.1% formic acid in acetonitrile as solvent B. A step gradient program was developed for the best separation of all metabolites: 0–0.5 min 20% B and 0.5–3.0 min 100% B. The column was then reconditioned to 20% B for 0.5 min. The total run time for analysis was 3.5 min and the injection volume 5 μl. For analysis of NAPEs of PEA and SEA and their deuterated analogs, a reversed phase T3 column (50 × 2.1 mm inner diameter, 1.8 μm particle size, maintained at 50°C; Waters Inc.) was used with a constant flow rate of 0.4 ml/min. The mobile phase consisted of 10 mM ammonium formate in acetonitrile:water (60:40) as solvent A and 10 mM ammonium formate in acetonitrile:isopropanol (10:90) as solvent B. A step gradient program was developed for the best separation of all metabolites: 0–0.5 min 50% B, 0.5–3.5 min 50 to 100% B, 3.5–4.5 min 100% B, and 4.5–5.0 min 100 to 50% B. The column was then reconditioned to 50% B for 1 min. The total run time for analysis was 6 min and the injection volume 5 μl. Lastly, conditions for analysis of NAPEs of OEA were the same as above for the other NAPEs with little modifications: constant flow rate of 0.35 ml/min; a step gradient of 0–0.5 min 30% B, 0.5–6.0 min 30 to 100% B, 6.0–7.0 min 100% B, 7.0–7.1 min 100 to 50% B, and reconditioned column to 30% B for 1.9 min; and total run time for analysis of 9 min. For both NAEs and NAPEs, the mass spectrometer was operated in the positive ESI mode, the capillary voltage was set at 3 kV, the cone voltage was set at 20 V for all transitions, and analytes were quantified by multiple reactions monitoring (MRM). The complete panel of source parameters and MRM transitions are reported in the datasheet of the Supplementary Material (Supplementary Tables S1, S2). The source temperature was set to 120°C. Desolvation gas and cone gas (N_2_) flows were set to 800 and 50 l/h, respectively. Desolvation temperature was set to 450°C. Data were acquired by MassLynx software and quantified by TargetLynx software. Calibration curves (0.1 to 100 nM range for all compounds) were constructed by plotting the analyte to IS peak areas ratio versus the corresponding analyte concentration using weighted (1/×) least square regression analysis.

### Determination of FAAH Activity

The initial segment of the anterior intestine (the other transversal half) and the other longitudinal half of hypothalamus were weighed, homogenized in ice-cold Tris–HCl buffer (20 mM, pH 7.4) containing 0.32 M sucrose, and centrifuged at 1000 × *g* for 10 min at 4°C. Supernatants were collected and protein concentrations determined using a bicinchoninic acid (BCA) assay kit (Pierce, Rockford, IL, United States). To measure FAAH activity, 0.5 ml of Tris–HCl buffer (50 mM, pH 7.4) containing fatty acid-free bovine serum albumin (0.05%), tissue homogenates (50 μg of protein), 10 μM AEA and AEA-(ethanolamine-^3^H) (20,000 cpm, specific activity 60 Ci/mmol; American Radiolabeled Chemicals, St Louis, MO, United States) were incubated at 37°C for 30 min. Reactions were stopped with 1 ml methanol:chloroform (1:1), centrifuged at 1400 × *g* for 10 min at 4°C, and radioactivity was measured in the aqueous phase by liquid scintillation counting in MicroBeta LumiJET system (Perkin Elmer Inc., Waltham, MA, United States).

### Gene Expression Analysis

Total RNA from the initial segment of the intestinal bulb (3 mm), the distal segment of the anterior intestine (5 mm), and the other liver aliquot was isolated using TRI^^®^^ Reagent (Sigma-Aldrich) and treated with RQ1 RNase-Free DNase (Promega, Madison, United States) according to the manufacturer’s instructions. Then, an aliquot of total RNA (0.1 μg of intestinal bulb and anterior intestine, or 0.3 μg of liver) was reverse transcribed into cDNA in a 25 μl reaction volume using random primers (Invitrogen, Carlsbad, United States), RNase inhibitor (Promega) and SuperScript II Reverse Transcriptase (Invitrogen). The reverse transcription reaction conditions consisted of an initial step at 25°C for 10 min, an extension at 42°C for 50 min, and a denaturalization step at 70°C for 15 min. Real-Time quantitative PCRs (RT-qPCRs) were carried out by duplicate in a CFX96^TM^ Real-Time System (Bio-Rad Laboratories, Hercules, United States), using iTaq^TM^ Universal SYBR^^®^^ Green Supermix (Bio-Rad Laboratories) in a 96-well plate loaded with 1 μl of cDNA and a final concentration of 0.5 μM of each forward and reverse primers in a final volume of 10 μl. Each PCR run also included a 4-point serial standard curve, non-retrotranscribed RNA (as positive control) and water (as negative control). The RT-qPCR cycling conditions consisted of an initial denaturation at 95°C for 30 s and 40 cycles of a two-step amplification program (95°C for 5 s and 60°C for 30 s). A melting curve was systematically monitored (temperature gradient at 0.5°C/5 s from 70 to 90°C) at the end of each run to confirm the specificity of the amplification reaction. The Gene Data Bank reference numbers and the primers (Sigma-Aldrich) sequences employed for target genes (*nape-pld*, *faah ppar*α, *bmal1a*, and *rev-erb*α) and the reference gene (*ef-1α*) are shown in Table 1. The 2^-ΔΔCt^ method (Livak and Schmittgen, 2001) was used to determine the relative mRNA expression (fold change). Data obtained were normalized to the group with the lowest expression in each gene.

**Table 1:** Accession numbers and primers sequences of the genes employed in the RT-qPCR assays.

	Accession			Product
Gene	number	Primer sequence 5′ → 3′		(bp)
*nape-pld*	MH638307	Forward	TGCTCGCTTTGGTTCCAGT	115
		Reverse	AATGCAGTTTCCCACCCAC	
*faah*	HM231167.1	Forward	TTGGAGGAGGAGGCTCTTTG	115
		Reverse	CCACTGCAATGAGGATAAGTGC	
*pparα*	AY198322.1	Forward	CCATCCCGACAACGAGTTCC	121
		Reverse	CAGCGACGTGTCTTCTGTCT	
*bmal1a*	KF840401	Forward	AGATTCTGTTCGTCTCGGAG	161
		Reverse	ATCGATGAGTCGTTCCCGTG	
*rev*-erbα	KU242427.1	Forward	CGTTCATCTCAGGCACCACT	166
		Reverse	AACTGACCTGCAGACACCAG	
*ef-1α*	AB056104	Forward	CCCTGGCCACAGAGATTTCA	101
		Reverse	CAGCCTCGAACTCACCAACA	

*Nape-pld, N-acyl phosphatidylethanolamine-specific phospholipase D; faah, fatty acid amidohydrolase; pparα, peroxisome proliferator-activated receptor α; bmal1a, brain and muscle ARNT-like 1a; rev-erbα, nuclear receptor subfamily 1 group D member 1 (NR1D1); ef-1α, elongation factor-1α*.

### Data Analysis

All studied parameters were first analyzed by One-way ANOVA followed by the *post hoc* Student–Newman–Keuls (SNK) test (using SigmaPlot 12.0 statistics package). When necessary, data were transformed to logarithmic or square root scale to normalize and to obtain homoscedasticity. In addition, a Student *t*-test was performed to compare data from 1 h postprandial (sampling point ZT3) and 25 h fasting (ZT3b). A probability level of *p* < 0.05 was considered statistically significant in all tests. Furthermore, the existence of daily (24-h) rhythms were determined by Cosinor analysis fitting the data to a sinusoidal function by the least squares method (Duggleby, 1981). The formula used was *f(t)* = *M*+*A*∗cos(*t*π/12-Φπ/12), where *f(t)* was the gene expression level at a given time, the mesor (*M*) is the mean value, *A* is the sinusoidal amplitude of oscillation, *t* is time in hours, and Φ is the acrophase (time of peak expression). Non-linear regression allows the estimation of *M*, *A*, and Φ, and their standard error (SE), being the SE based on the residual sum of squares in the least-squares fit (Duggleby, 1981; Delgado et al., 1993). Significance of Cosinor analysis was defined by the noise/signal of amplitude calculated from the ratio *SE(A)/A* (Nisembaum et al., 2012). Data were considered to display a daily rhythm if it had both *p* < 0.05 by ANOVA and *SE(A)/A* < 0.3 by Cosinor analysis.

## Results

**Figure 1:**
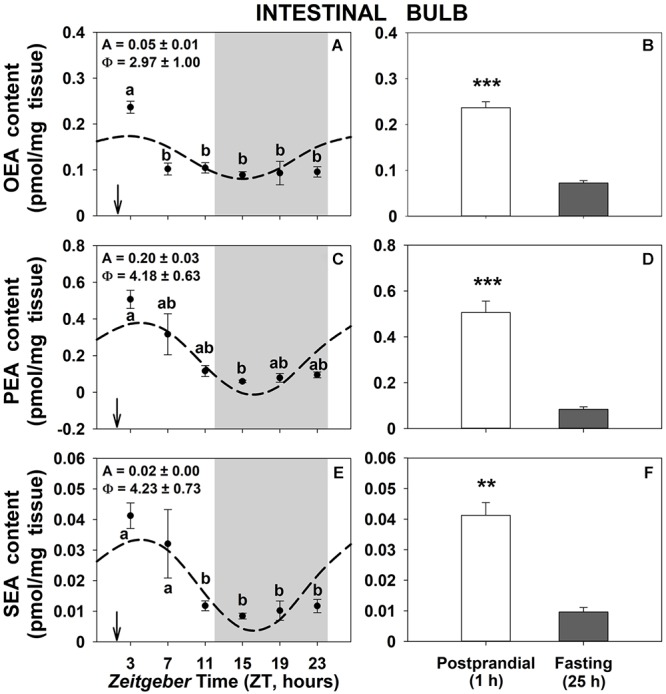
Daily variations of NAEs (OEA, PEA, and SEA) content in intestinal bulb of goldfish. **(A,C,E)**, profiles throughout a 24-h cycle. Gray area indicates the dark phase and arrows the feeding time (ZT2). *Different letters* indicate significant differences among groups (ANOVA, *p* < 0.05 and SNK *post hoc* test). Dashed lines represent periodic sinusoidal functions when rhythms are significant [Cosinor, *SE(A)/A* < 0.3; and ANOVA, *p* < 0.05]. Amplitude (A, fold change) and acrophase (Φ, h) values of the rhythms are shown in the top-left of the graphs. **(B,D,F)**, comparison between 1 h postprandial and 25 h fasting. *Asterisks* indicate significant differences by Student *t*-test analysis (^∗∗^*p* < 0.01, ^∗∗∗^*p* < 0.001). Data are shown as mean ± SEM (*n* = 6–7).

Daily patterns of OEA, PEA, and SEA levels in intestinal bulb of goldfish are shown in Figure 1. All three NAEs displayed significant rhythms with low amplitudes and the acrophase (time of the day with maximum levels) 3–4 h after mealtime (Figure 1A,C,E). The content of the three NAEs was 3–4 fold higher in the 1-h postprandial fishes than in 25-h fasting ones (Figure 1B,D,F). Similar results were obtained in the other two gastrointestinal tissues (Supplementary Figures S1, S3), being rhythmic PEA and SEA in the anterior intestine and OEA in the liver. As for intestinal bulb, the content of NAEs was higher in the 1-h postprandial than in 25-h fasting fish in both peripheral tissues, although the trend not was statistically significant in the case of SEA in the liver (Supplementary Figure S3F).

**Figure 2:**
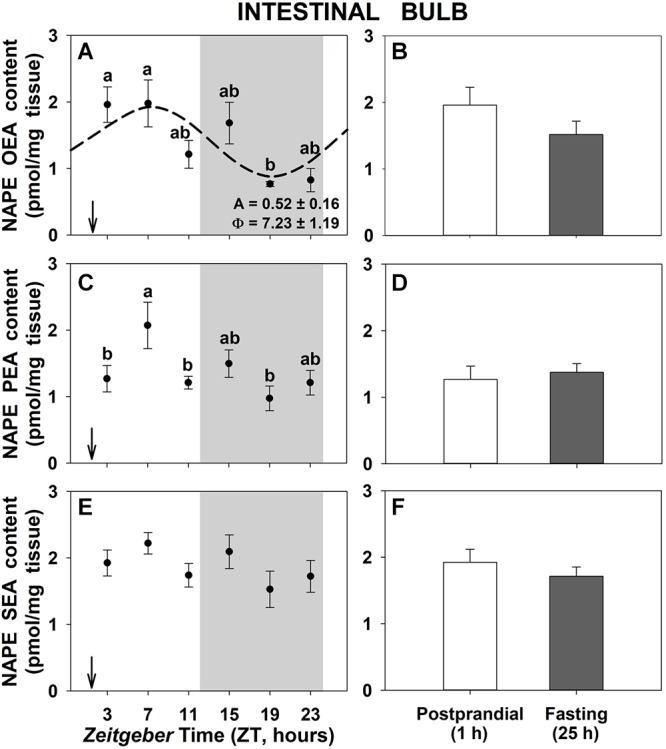
Daily variations of NAPEs content in intestinal bulb of goldfish. **(A,C,E)**, profiles throughout a 24-h cycle. Gray area indicates the dark phase and arrows the feeding time (ZT2). *Different letters* indicate significant differences among groups (ANOVA, *p* < 0.05 and SNK *post hoc* test). Dashed line represents periodic sinusoidal function when rhythm is significant [Cosinor, *SE(A)/A* < 0.3; and ANOVA, *p* < 0.05]. Amplitude (A, fold change) and acrophase (Φ, h) values of the rhythm are shown in the bottom-right of the graphs. **(B,D,F)**, comparison between 1 h postprandial and 25 h fasting. Data are shown as mean ± SEM (*n* = 6–7).

Regarding the precursors of NAEs, the daily profiles of NAPEs corresponding to each NAE in intestinal bulb of goldfish are shown in Figure 2. We can observe daily variations in the content of NAPE of OEA and PEA in intestinal bulb with the maximum levels occurring during the day time and the lowest in the middle of the night (Figure 2A,C,E). Only the NAPE of OEA displayed a significant rhythm (Figure 2A). On the other hand, 25-h fasting did not modify the total amount of NAPEs respect to 1-h postprandial (Figure 2B,D,F). In the other two gastrointestinal tissues, the anterior intestine and the liver (Supplementary Figures S2, S4, respectively), no significant daily oscillations of NAPEs were found and only the PEA-NAPE showed a significant higher content in the 1-h postprandial compared to 25-h fasting animals.

**Figure 3:**
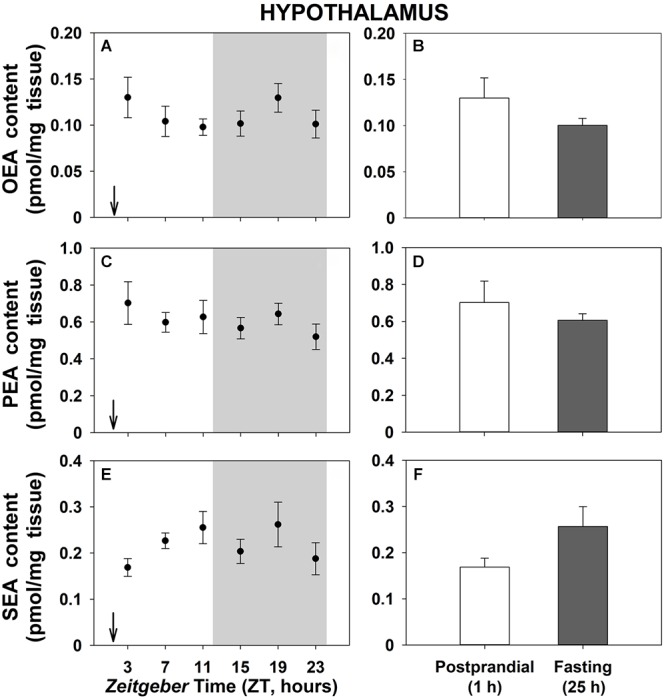
Daily variations of NAEs (OEA, PEA, and SEA) content in hypothalamus of goldfish. **(A,C,E)**, profiles throughout a 24-h cycle. Gray area indicates the dark phase and arrows the feeding time (ZT2). **(B,D,F)**, comparison between 1 h postprandial and 25 h fasting. Data are shown as mean ± SEM (*n* = 6–7).

Daily patterns of NAEs levels in the hypothalamus are shown in Figure 3. Contrary to gastrointestinal tissues, no significant differences were found neither throughout the 24-h cycle (Figure 3A,C,E) nor when 1-h postprandial and 25-h fasting animals were compared (Figure 3B,D,F). Similar results were obtained in the other studied brain tissue, the telencephalon (Supplementary Figure S5), except for SEA that showed daily oscillations with the lowest content at the beginning of the dark phase, although rhythmicity did not reach the threshold of significance (Supplementary Figure S5E).

**Figure 4:**
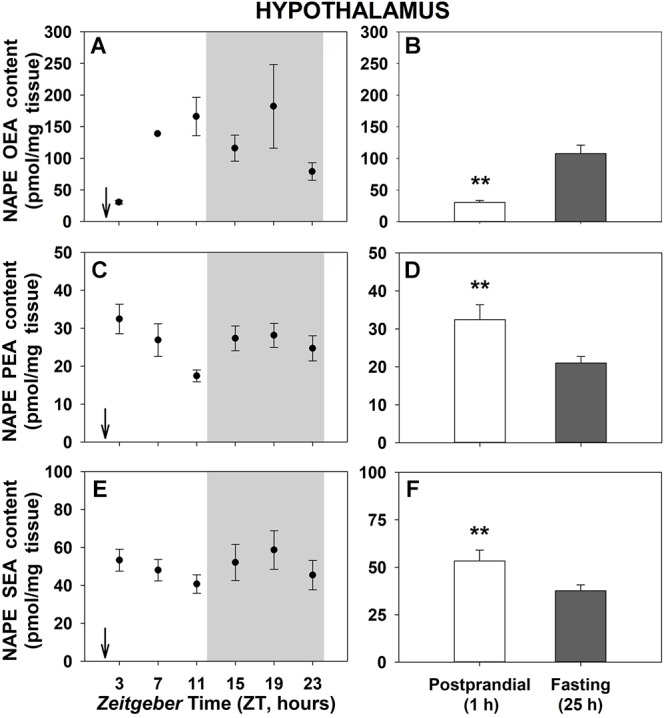
Daily variations of NAPEs content in hypothalamus of goldfish. **(A,C,E)**, profiles throughout a 24-h cycle. Gray area indicates the dark phase and arrows the feeding time (ZT2). **(B,D,F)**, comparison between 1 h postprandial and 25 h fasting. *Asterisks* indicate significant differences by Student *t*-test analysis (^∗∗^*p* < 0.01). Data are shown as mean ± SEM (*n* = 6–7).

Figure 4 shows the daily profiles of the NAPEs corresponding to each NAE in the hypothalamus of goldfish. No significant oscillations were obtained throughout the 24-h cycle (Figure 4A,C,E). However, significant differences between 1-h postprandial and 25-h fasting were noticed for all NAPEs in this encephalic tissue (Figure 4B,D,F). While hypothalamic levels of NAPE of OEA was increased by fasting, both NAPEs of PEA and SEA were decreased. Obtained results in the other central tissue, the telencephalon (Supplementary Figure S6), showed daily oscillations in all NAPEs with only a significant 24-h rhythm in the NAPE of OEA (Supplementary Figures S6A,C,E), but did not exhibit feeding-induced changes in the levels of any studied NAPEs (Supplementary Figures S6B,D,F).

**Figure 5:**
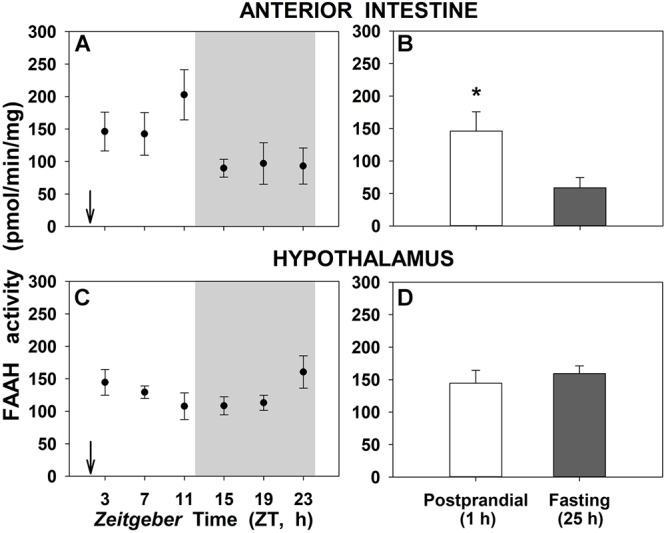
Daily variations of FAAH activity in anterior intestine **(A,B)** and hypothalamus **(C,D)** of goldfish. **(A,C)**, profiles throughout a 24-h cycle. Gray area indicates the dark phase and arrows the feeding time (ZT2). **(B,D)**, comparison between 1 h postprandial and 25 h fasting. *Asterisks* indicate significant differences by Student *t*-test analysis (^∗^*p* < 0.05). Data are shown as mean ± SEM (*n* = 6–7).

The enzymatic activity of FAAH, the degradation enzyme of NAEs, in anterior intestine and hypothalamus of goldfish is shown in Figure 5. There are no daily changes in none of the studied tissues (Figure 5A,C). Instead, a significant threefold increase were noted in 1-h postprandial fish respect to 25-h fasting in the anterior intestine, while FAAH activity in hypothalamus remained unchanged with feeding (Figure 5B,D, respectively).

**Figure 6:**
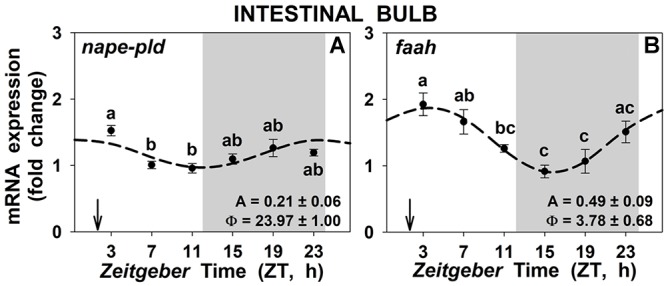
Daily rhythms of *nape-pld* **(A)** and *faah* **(B)** expression in intestinal bulb of goldfish. Gray area indicates the dark phase and arrows the feeding time (ZT2). Data are shown as mean ± SEM (*n* = 6–7) in relative units (2^-ΔΔCt^ method). *Different letters* indicate significant differences among groups (ANOVA, *p* < 0.05 and SNK *post hoc* test). Dashed lines represent periodic sinusoidal functions when rhythms are significant [Cosinor, *SE(A)/A* < 0.3; and ANOVA, *p* < 0.05]. Amplitude (A, fold change) and acrophase (Φ, h) values of the rhythms are shown in the bottom-right of the graphs.

The mRNA abundance of *nape-pld* and *faah* genes (which codify for the NAEs synthesis and degradation enzymes, respectively) in intestinal bulb presented significant daily rhythms (Figure 6), with low amplitudes, and no differences were found between 1 h-posprandrial and 25 h fasting (data not shown). The acrophase of the *nape-pld* gene took place in the interphase dark-light, 2 h before the mealtime, while the acrophase of *faah* gene took place around ZT4, 2 h after the mealtime. A similar pattern was observed for *nape-pld* in liver but not in the anterior intestine (Supplementary Figures S7A,C) and for *faah* in both anterior intestine and liver (Supplementary Figures S7B,D), although such daily differences were not associated with significant rhythms.

**Figure 7:**
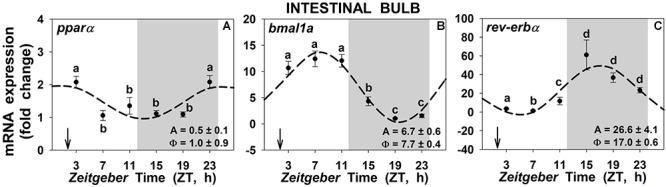
Daily rhythms of *pparα* **(A)**, *bmal1a* **(B)** and *rev*-erbα **(C)** expression in intestinal bulb of goldfish. Gray area indicates the dark phase and arrows the feeding time (ZT2). Data are shown as mean ± SEM (*n* = 6–7) in relative units (2^-ΔΔCt^ method). *Different letters* indicate significant differences among groups (ANOVA, *p* < 0.05, and SNK *post hoc* test). Dashed lines represent periodic sinusoidal functions when rhythms are significant [Cosinor, *SE(A)/A* < 0.3; and ANOVA, *p* < 0.05]. Amplitude (A, fold change) and acrophase (Φ, h) values of the rhythms are shown in the bottom-right of the graphs.

Daily significant expression rhythms of the NAEs receptor (*pparα*) and of the two studied clock genes (*bmal1a* and *rev-erb*α) were found in the intestinal bulb of goldfish (Figure 7). The acrophase of *pparα* (Figure 7A) took place at ZT1, 1 h before mealtime and 1 h after the onset of the light, while the acrophase of *bmal1a* (Figure 7B) took place around ZT8, 6 h after mealtime. The acrophase of *rev*-erbα (Figure 7C) occurs at ZT17, almost in the middle of the scotophase. With regards to the other two peripheral tissues, the anterior intestine and the liver (Supplementary Figure S8), the circadian rhythms were maintained as for the intestinal bulb, with comparable acrophases and amplitudes.

## Discussion

Our results show for the first time in a fish species the existence of all components of NAEs system: OEA, PEA, SEA, their precursors, enzymes of synthesis and degradation, and the receptor PPARα. Endogenous levels of NAEs and NAPEs found in gastrointestinal and brain tissues in goldfish are similar to that previously reported in other vertebrates, although very few species have been studied (Astarita et al., 2006; Murillo-Rodriguez et al., 2006; Fu et al., 2007; Guijarro et al., 2010; Liedhegner et al., 2014). These data suggest that these bioactive lipids may be widespread across vertebrate groups.

NAEs (OEA, PEA, and SEA) exhibit daily variations in the goldfish gastrointestinal tissues, which seemed to follow daily rhythmic patterns, being mainly driven by food intake. In fact, the most interesting result of this study is a pronounced and rapid postprandial increase in the content of the three NAEs analyzed in intestinal bulb, anterior intestine, and liver of goldfish, compared to levels found in 25-h fasting fish. These results agree with the OEA formation promoted by feeding in the small intestine previously found in mammals, reptiles, and goldfish (Astarita et al., 2006; Piomelli, 2013; Tinoco et al., 2014; Bowen et al., 2017). Both rat and Burmese python also exhibit these fasting/refeeding-induced changes in the intestinal content of PEA and SEA (Astarita et al., 2006; Petersen et al., 2006; Diep et al., 2011). These periprandial fluctuations in the gastrointestinal content of NAEs suggest that this family of bioactive lipids may contribute to the regulation of feeding behavior in vertebrates, possibly acting as satiety signals, since intestinal levels are elevated in the post-ingestive state. Pharmacological acute studies in rodents support this idea, demonstrating anorectic effects of OEA, PEA and SEA in rats and mice, which are peripherally mediated (Rodríguez de Fonseca et al., 2001; Terrazzino et al., 2004; Piomelli, 2013). Similar results have also been found in goldfish, where a reduction in food intake was produced after intraperitoneal administration of OEA (Tinoco et al., 2014). Thus, all results support that NAEs are involved in feeding regulation, acting as short-term anorectic signals.

The postprandial increase of NAEs in intestinal tissues may be due to an increase of their precursors and/or changes in the activity of enzymes involved in their synthesis and degradation (Fu et al., 2007; Bowen et al., 2017). Some studies in mammals have suggested that NAEs levels are regulated in intestinal tissue in parallel with the formation of their precursor molecules, the NAPEs (Petersen et al., 2006; Fu et al., 2007; Gillum et al., 2008). In addition, Fu et al. (2007) found that feeding stimulates OEA mobilization in duodenum and jejunum by increasing activity and expression of NAPE-PLD. However, most data in the present study indicate that there are no feeding-related differences in the gastrointestinal content of NAPEs nor relative mRNA expression of *nape-pld*, even though significant changes in NAEs levels were observed during fasting/feeding cycles. Some hypotheses could explain this lack of differences in precursors and synthesis enzyme in fish. On one hand, it should also be taken into account that, in addition to the direct hydrolysis from NAPEs to NAEs by NAPE-PLD, other multistep pathways of NAEs formation exist (Hussain et al., 2017; Inoue et al., 2017). These alternative pathways involve intermediate molecules, such as glycerophospho-NAE, lyso-N-acyl-phosphatidylethanolamine, or phospho-*N*-acylethanolamine, and a possible decrease of these intermediates could explain the increase of NAEs levels observed in gastrointestinal tissues in goldfish, without feeding-related modifications in levels of NAPEs and the NAPE-PLD enzyme. In addition, Lin et al. (2018) demonstrated that dietary fatty acids can modulate tissue NAEs levels in the absence of NAPE-PLD, which suggest that NAPE-PLD is not necessary for NAEs synthesis, thereby highlighting the important role of alternative pathways in maintaining NAEs levels. Other possibility is that the NAEs regulation by feeding occurs at level of the degradation enzyme, since a decrease in FAAH activity and expression in rodents’ intestine was found after feeding (Fu et al., 2007), being responsible, at least in part, of feeding-induced OEA increase. However, this expected negative correlation between NAEs concentration and FAAH was not found in goldfish tissues. Other studies in mammals have indicated that FAAH activity has a small contribution in NAEs levels, suggesting the existence of other amidases, such as NAAA, also responsible for NAEs metabolism (Borrelli and Izzo, 2009; Liedhegner et al., 2014; Bowen et al., 2017). Thus, other forms of amidase not yet characterized in fish could also contribute to the postprandially NAEs-increased levels in goldfish. The postfeeding increase in the expression and activity of FAAH in some gastrointestinal tissues of goldfish could be a physiological response to the rise in NAEs levels due to the upregulation of any enzyme involved in the formation of NAEs, as it has been previously suggested in rats (Diep et al., 2011).

Less clear is the role of NAEs at central level and very few studies have examined daily changes in the brain content of NAEs. In the present study, the NAEs content in the goldfish hypothalamus and telencephalon did not display significant rhythms. Similar results were found in mice, where daily oscillations were not detected in hypothalamic content of OEA and PEA, although these NAEs exhibited diverse daily rhythms in other brain regions, such as cerebellum, amygdala, and hippocampus, suggesting that these daily changes in NAEs are brain region-specific (Liedhegner et al., 2014). Controversial results have been reported in rats. While no effect of daytime (photophase versus scotophase) was found in OEA content in various encephalic tissues in rats (cerebellum, hippocampus, hypothalamus, thalamus, cortex, striatum, and brainstem; Guijarro et al., 2010), diurnal variations of OEA and PEA were detected in pons, hippocampus, and hypothalamus in the same species (Murillo-Rodriguez et al., 2006). Independently of existence or not of daily modifications in the NAEs content in different encephalic tissues, it has been suggested that the brain content of these compounds seem to be feeding independent. Thus, OEA did not respond to food deprivation in different rat brain tissues, including structures involved in the control of feeding, such as hypothalamus, thalamus, cortex, striatum, and brainstem (Fu et al., 2007; Izzo et al., 2010). Similarly, no differences in NAEs content in hypothalamus and telencephalon were observed between fasting and feeding states in goldfish. This feeding-independent regulation of NAEs in the brain suggests that nutritional status could be regulating NAEs mobilization in a tissue-specific manner only at gastrointestinal level. In addition, it would support the above discussed idea that NAEs play a role in the feeding regulation at peripheral level. Nevertheless, it cannot be ruled out that NAEs play other physiological roles at the brain level, although they have not been investigated in fish yet.

Although NAPEs have been considered for a long time as simply phospholipid precursors of the NAEs, the increasing body of evidence in mammals has suggested that NAPEs also seem to be bioactive molecules that are involved in several physiological functions, without the involvement of NAEs (Coulon et al., 2012; Romano et al., 2015). Particularly, it has been demonstrated that hypothalamic administration of C16:0 NAPE (N-palmitoyl-phosphatidylethanolamine, the most abundant plasmatic NAPE) decreases food intake in rats, and its effect does not seem to be mediated by a NAPE metabolite (NAE) (Gillum et al., 2008; Wellner et al., 2011). In the present study, we have found postprandial changes in NAPEs at hypothalamic level in goldfish, which could suggest a possible involvement of these NAPEs in the central regulation of feeding. Although, because OEA-NAPE content decreases and PEA- and SEA-NAPE content increases after feeding, the interpretation of these results is difficult. Further studies must to be performed in order to clarify the exact role of NAPEs in fish brain and if they are signaling lipids able to control important biological functions on their own.

The expression of the NAEs receptor, *pparα*, displayed a clear daily rhythm in all the studied gastrointestinal tissues, suggesting a possible rhythmicity in the functions of NAEs. In nocturnal rodents, *pparα* expression in liver increases during the daytime, having its maximum at the beginning of the night (Yang et al., 2006; Chen et al., 2010; Wang et al., 2014). Our data indicate that in the diurnal-species goldfish, *pparα* expression rises during the nighttime peaking in the early morning (1 h before feeding) in intestinal tissues and liver, similar to that reported in the liver of sea bream, another diurnal fish (Paredes et al., 2014). In both nocturnal and diurnal animals, the PPARα is upregulated during the rest phase, which coincides with the fasting state of the animals (Liu et al., 2014). During this fasting state, animals obtain energy from increasing the hepatic fatty acid oxidation with the synthesis of ketone bodies (Ribas-Latre and Eckel-Mahan, 2016). In fact, it has been demonstrated in mammals that PPARα stimulates fatty acid oxidation and lipid catabolism (Charoensuksai and Xu, 2010; Chen and Yang, 2014; Liu et al., 2014). In addition, *bmal1a* is also rhythmic increasing after feeding, supporting its role as a lipogenic factor in mammals (Shimba et al., 2005, 2011; Zhang et al., 2014), and also in goldfish (Gómez-Boronat et al., 2016). Moreover, PPARα has been largely proposed as a link between lipid metabolism and circadian system in mammals (Yang et al., 2006; Chen and Yang, 2014; Ribas-Latre and Eckel-Mahan, 2016), in which circadian rhythms of PPARα are essential for the temporal coordination of genes involved in energy and metabolic process (Charoensuksai and Xu, 2010; Chen and Yang, 2014; Ribas-Latre and Eckel-Mahan, 2016). Thus, it is widely known that PPARα directly regulates the transcription of *bmal1* and *rev*-erbα via binding to the peroxisome proliferator response element (PPRE) sites in their respective promoter regions. In addition, BMAL1 induces *pparα* and *rev*-erbα by binding to an E-box rich region in their respective promoters (Canaple et al., 2006; Charoensuksai and Xu, 2010; Chen and Yang, 2014; Lecarpentier et al., 2014). In accordance with this regulatory loop, in the three gastrointestinal tissues of goldfish here analyzed (intestinal bulb, anterior intestine, and liver), the acrophases of *pparα* and *bmal1a* are ∼8-h shifted, as previously reported in mammals (8–14-h shift; Canaple et al., 2006; Yang et al., 2006; Chen et al., 2010). The existence of the expected daily rhythms in all studied clock genes supports the idea that clocks in gastrointestinal tissues are functional.

In summary, the identification in goldfish of the NAEs system, including precursors, enzymes of synthesis and degradation and receptor, suggests that this endogenous system can be an important pathway for physiological functions as regulation of energy homeostasis in fish, as it is mammals. The gastrointestinal regulation of NAEs levels by the fed and fasted metabolic states supports that NAEs are involved in the feeding regulation, acting as a peripheral satiety signal. In addition, the present results are in agreement with a putative role of PPARα as a functional link between the circadian clock and lipid metabolism in fish.

## Ethics Statement

This study was carried out in accordance with the recommendations of Guidelines of the European Union Council (UE63/2010) and the Spanish Government (RD53/2013). The protocol was approved by the Animal Experimentation Committee of Complutense University (O.H.-UCM-25-2014) and the Community of Madrid (PROEX 107/14).

## Author Contributions

MG-B, EI, MD, and NdP conceived and designed the experiments, carried out sampling, and interpreted findings. MG-B, EI, NdP, AA, and DP analyzed the samples. All authors drafted and revised the manuscript.

## Conflict of Interest Statement

The authors declare that the research was conducted in the absence of any commercial or financial relationships that could be construed as a potential conflict of interest. The reviewer NL declared a shared affiliation, with no collaboration, with one of the authors, DP, to the handling Editor at the time of review.
