# Supplementary material for: Diurnal Profiles of N-Acylethanolamines in Goldfish Brain and Gastrointestinal Tract: Possible Role of Feeding
Source: Front Neurosci. 2019 May 7;13:450. doi: 10.3389/fnins.2019.00450 (PMC6514144; doi:10.3389/fnins.2019.00450)
Supplement: Supplementary file 1 [file Table_1.DOCX]

**Table S1.** Complete panel of source parameters and MRM transitions of NAEs.

| **NAEs – MRM Transitions** | **Abbreviation used in the text** | **Retention Time (min)** | **Parent ion (m/z)** | **Daughter ion (m/z)** | **Collision Energy (V)** |
| --- | --- | --- | --- | --- | --- |
| Oleoylethanolamide (d18:1) | OEA | 2.70 | 326 | 62 | 20 |
| Oleoylethanolamide (d18:1) | OEA–d_4_ | 2.70 | 330 | 66 | 20 |
| Palmitoylethanolamide (d16:0) | PEA | 2.65 | 300 | 62 | 20 |
| Palmitoylethanolamide (d16:0) | PEA–d_4_ | 2.65 | 304 | 66 | 20 |
| Stearoylethanolamide (d18:0) | SEA | 2.90 | 328 | 62 | 20 |
| Stearoylethanolamide (d18:0) | SEA–d_3_ | 2.89 | 331 | 66 | 20 |
