# Supplementary material for: Diurnal Profiles of N-Acylethanolamines in Goldfish Brain and Gastrointestinal Tract: Possible Role of Feeding
Source: Front Neurosci. 2019 May 7;13:450. doi: 10.3389/fnins.2019.00450 (PMC6514144; doi:10.3389/fnins.2019.00450)
Supplement: Supplementary file 2 [file Table_2.DOCX]

**Table S2.** Complete panel of source parameters and MRM transitions of NAPEs.

| **NAPEs – MRM Transitions** | **Abbreviation used in the text** | **Retention Time (min)** | **Parent ion (m/z)** | **Daughter ion (m/z)** | **Collision Energy (V)** |
| --- | --- | --- | --- | --- | --- |
| 16:0-18:3-N18:1 NAPE | NAPE OEA | 5.38 | 994.7 | 308.3 | 20 |
| 16:0-20:4-N18:1 NAPE |  | 5.17 | 1020.7 | 308.3 | 20 |
| 16:0-22:5-N18:1 NAPE |  | 4.92 | 1046.7 | 308.3 | 20 |
| 16:0-22:6-N18:1 NAPE |  | 4.93 | 1044.7 | 308.3 | 20 |
| 18:0-18:1-N18:1 NAPE |  | 5.09 | 1026.8 | 308.3 | 20 |
| 18:0-18:3-N18:1 NAPE |  | 4.98 | 1022.7 | 308.3 | 20 |
| 18:0-20:4-N18:1 NAPE |  | 5.03 | 1048.7 | 308.3 | 20 |
| 18:0-22:5-N18:1 NAPE |  | 5.09 | 1074.8 | 308.3 | 20 |
| 18:0-22:6-N18:1 NAPE |  | 4.94 | 1072.7 | 308.3 | 20 |
| 18:1-18:1-N18:1 NAPE |  | 5.10 | 1024.7 | 308.3 | 20 |
| 18:1-18:2-N18:1 NAPE |  | 5.50 | 1022.7 | 308.3 | 20 |
| 18:1-18:3-N18:1 NAPE |  | 5.39 | 1020.7 | 308.3 | 20 |
| 18:1-20:4-N18:1 NAPE |  | 5.20 | 1046.7 | 308.3 | 20 |
| 18:1-22:5-N18:1 NAPE |  | 5.10 | 1072.7 | 308.3 | 20 |
| 18:1-22:6-N18:1 NAPE |  | 5.16 | 1070.7 | 308.3 | 20 |
| 16:0-16:0-N16:0 NAPE | NAPE PEA | 3.53 | 946.7 | 224.2 | 20 |
| 16:0-18:1-N16:0 NAPE |  | 3.53 | 972.7 | 224.2 | 20 |
| 18:0-22:6-N16:0 NAPE |  | 3.49 | 1046.7 | 224.2 | 20 |
| P18:0-22:6-N16:0 NAPE |  | 3.56 | 1077.7 | 224.2 | 20 |

**Table S2 (continue).** Complete panel of source parameters and MRM transitions of NAPEs.

| 16:0-18:1-N18:0 NAPE | NAPE SEA | 3.53 | 1000.8 | 252.3 | 20 |
| --- | --- | --- | --- | --- | --- |
| 18:0-20:4-N18:0 NAPE |  | 3.63 | 1050.8 | 252.3 | 20 |
| 16:0-22:6-N18:0 NAPE |  | 3.49 | 1046.7 | 252.3 | 20 |
| P16:0-22:6-N18:0 NAPE |  | 3.56 | 1077.7 | 252.3 | 20 |
| 18:0-22:6-N18:0 NAPE |  | 3.60 | 1074.8 | 252.3 | 20 |
| P18:0-22:6-N18:0 NAPE |  | 3.66 | 1105.7 | 252.3 | 20 |
| 16:0-16:0-N17:0 NAPE | IS NAPE | 3.55 | 960.7 | 238.3 | 20 |
| 18:0-22:6-N17:0 NAPE |  | 5.14 | 1056.7 | 238.3 | 20 |
