# Supplementary figures and images for: Diurnal Profiles of N-Acylethanolamines in Goldfish Brain and Gastrointestinal Tract: Possible Role of Feeding
Source: Front Neurosci. 2019 May 7;13:450. doi: 10.3389/fnins.2019.00450 (PMC6514144; doi:10.3389/fnins.2019.00450)

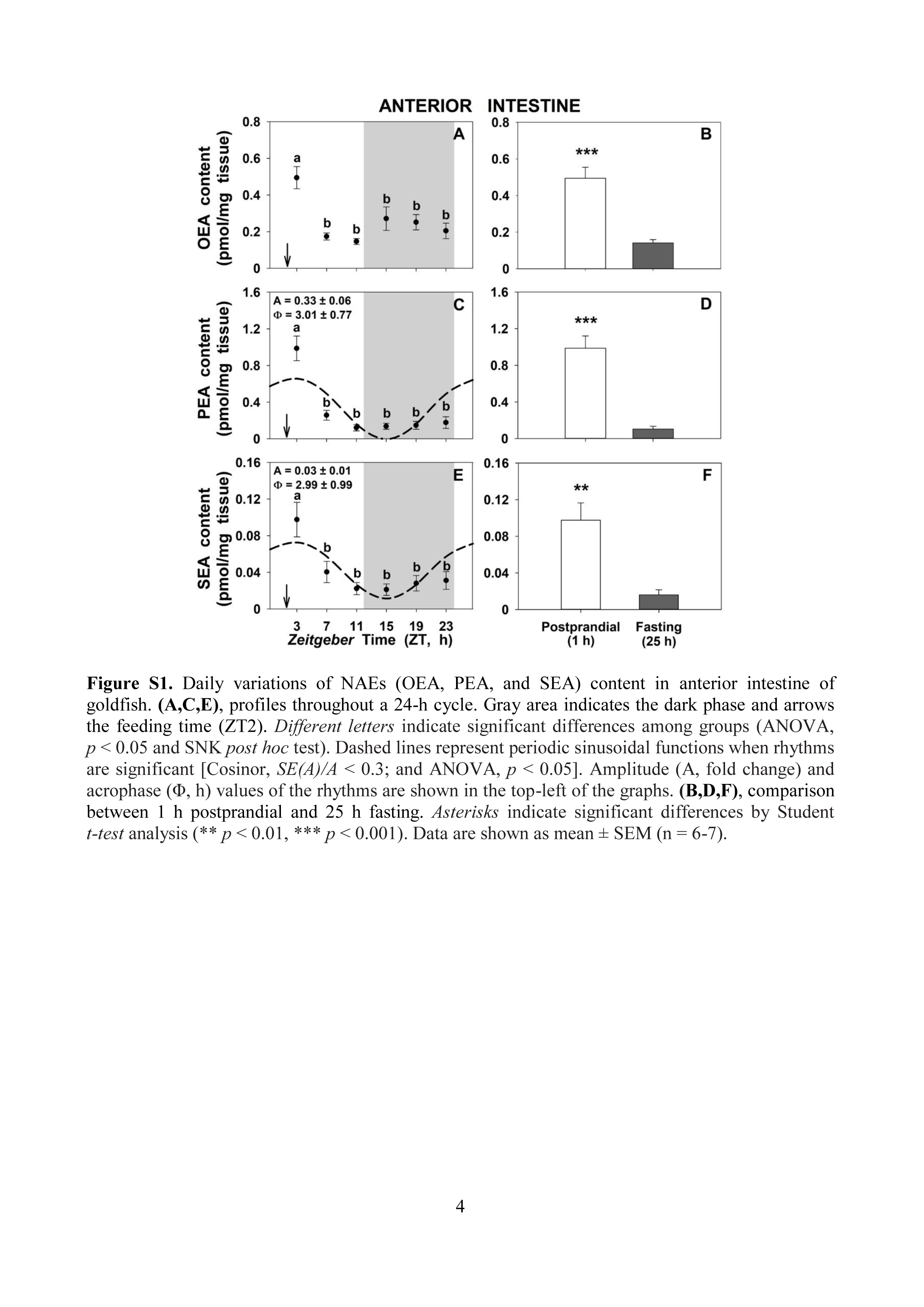

Supplement: Supplementary file 3 [file Image_1.jpg]

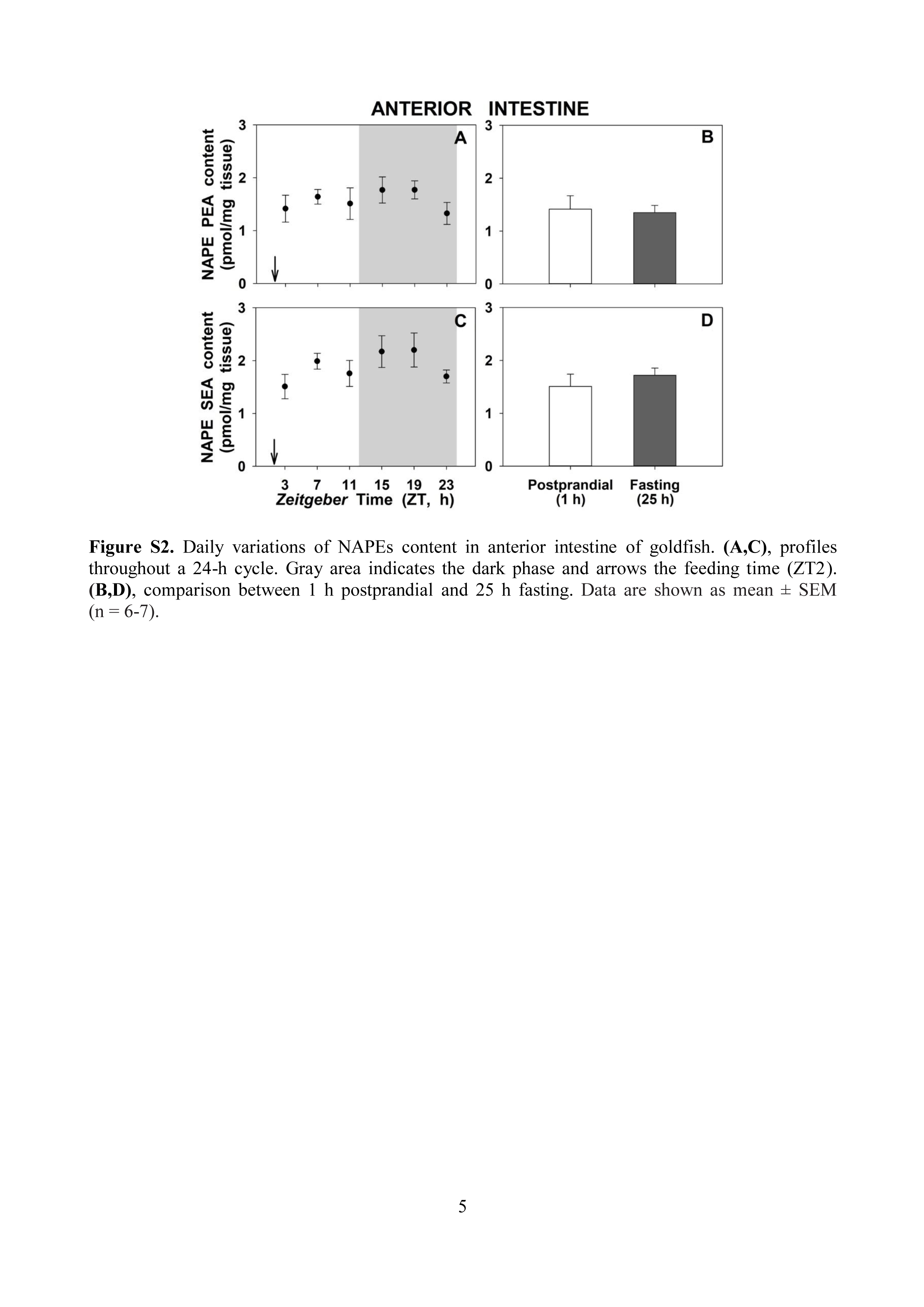

Supplement: Supplementary file 4 [file Image_2.jpg]

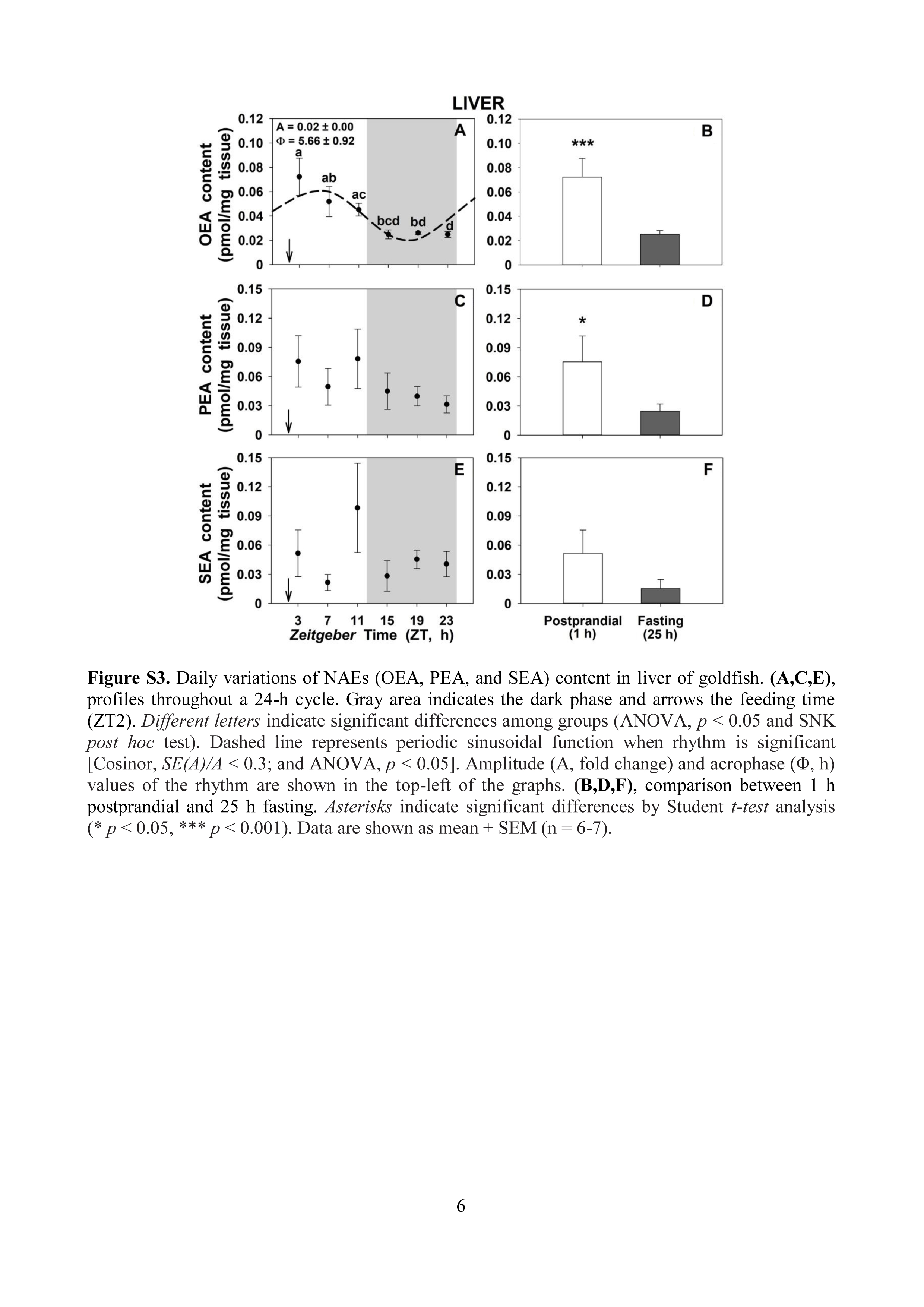

Supplement: Supplementary file 5 [file Image_3.jpg]

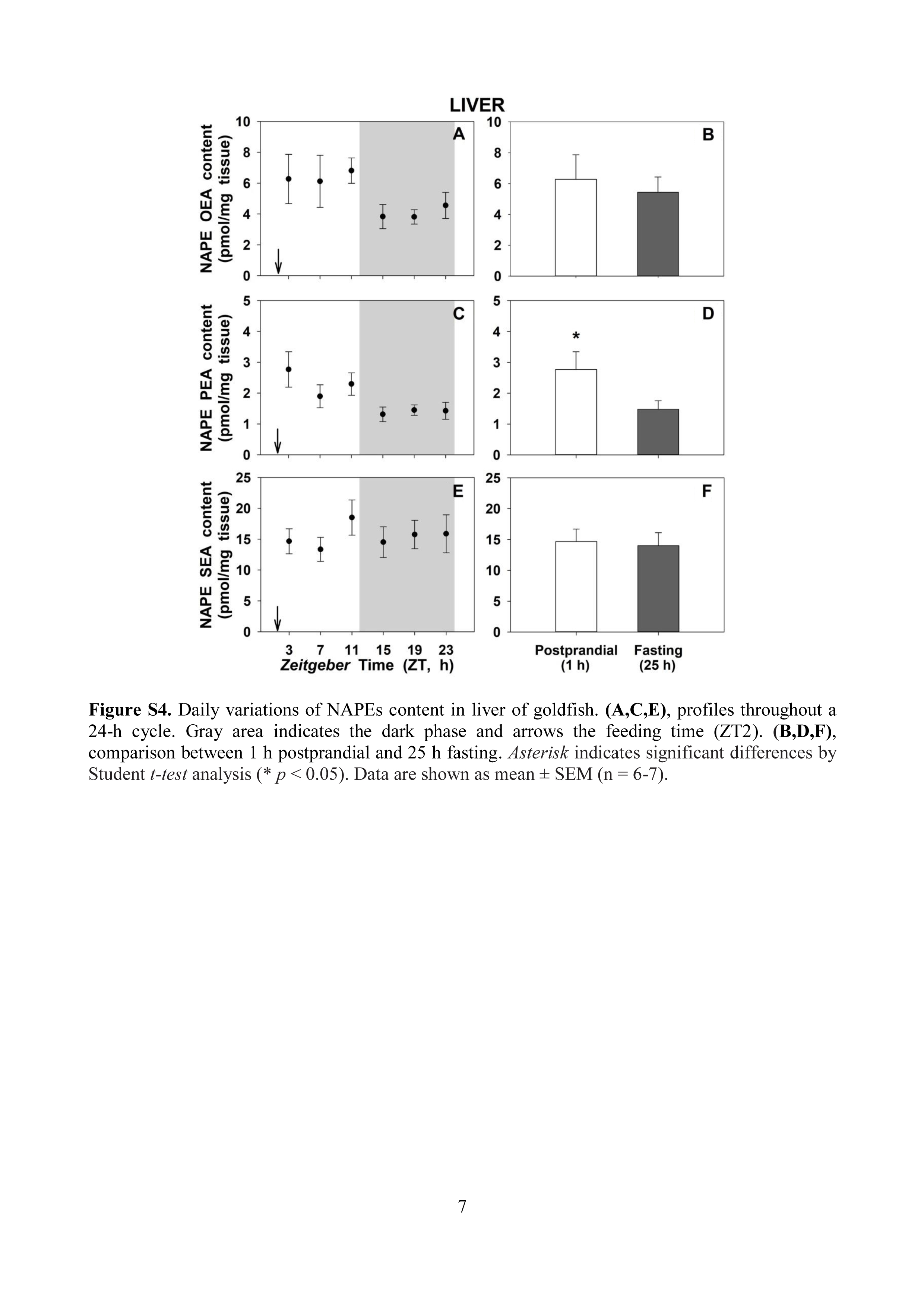

Supplement: Supplementary file 6 [file Image_4.jpg]

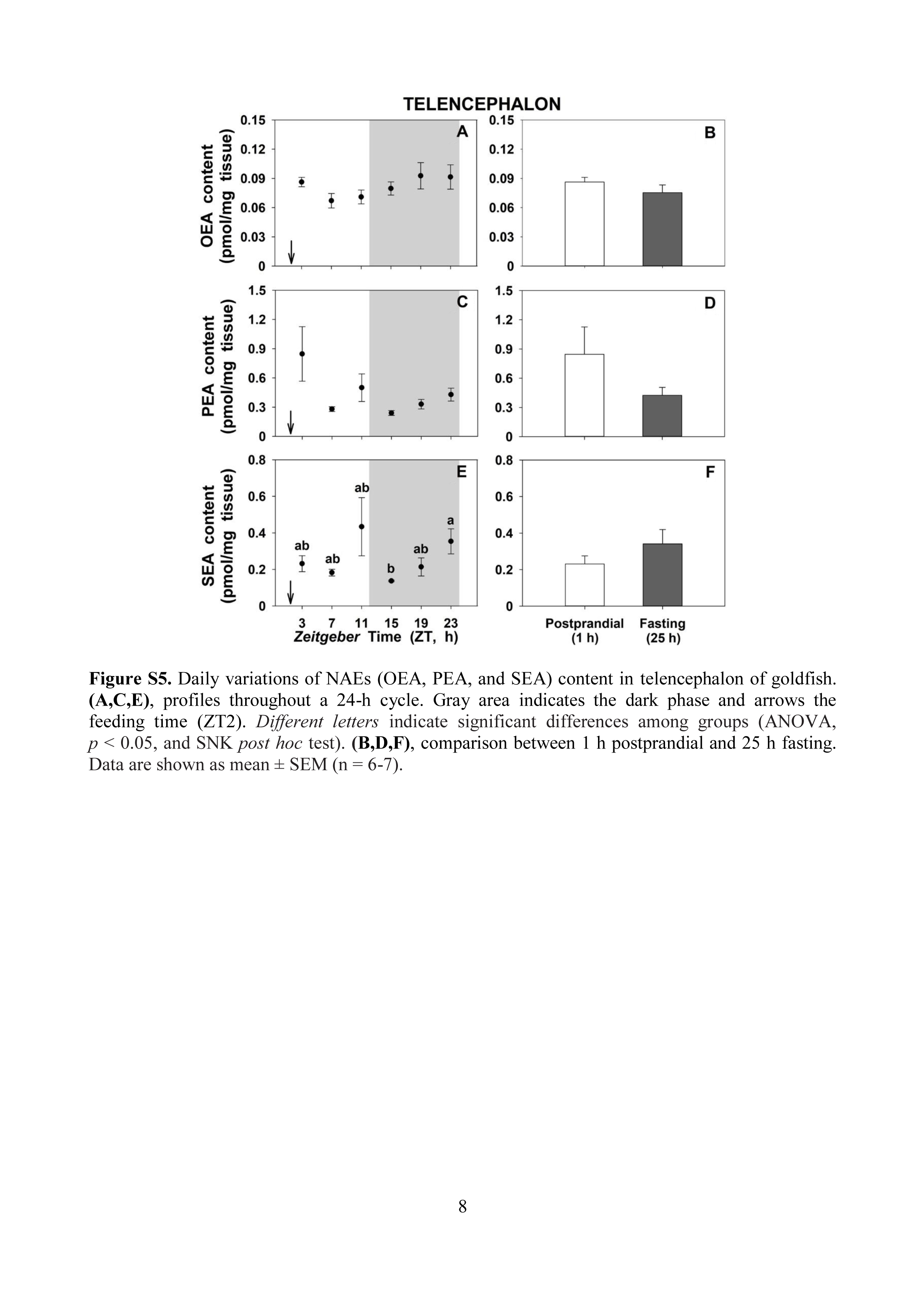

Supplement: Supplementary file 7 [file Image_5.jpg]

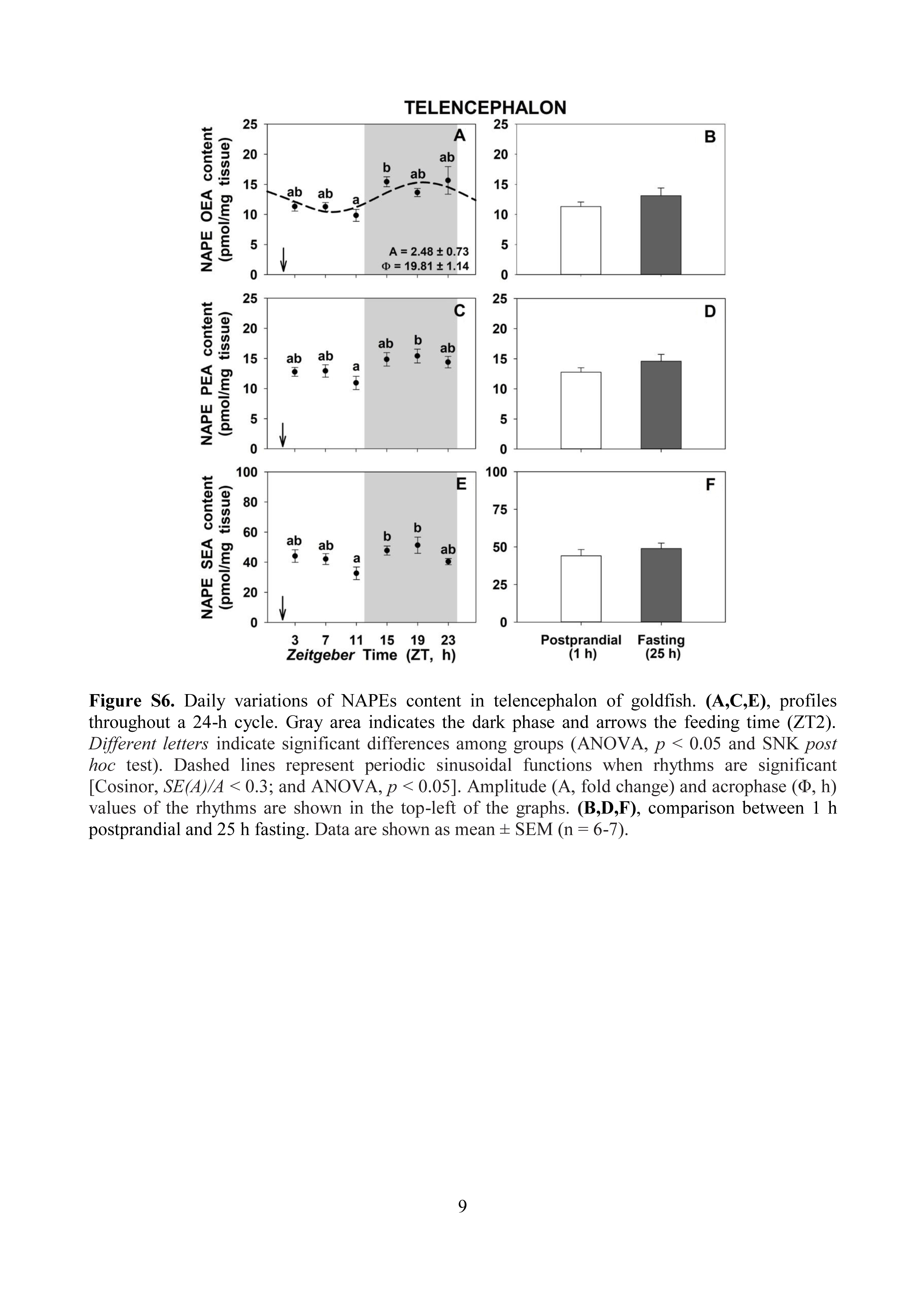

Supplement: Supplementary file 8 [file Image_6.jpg]

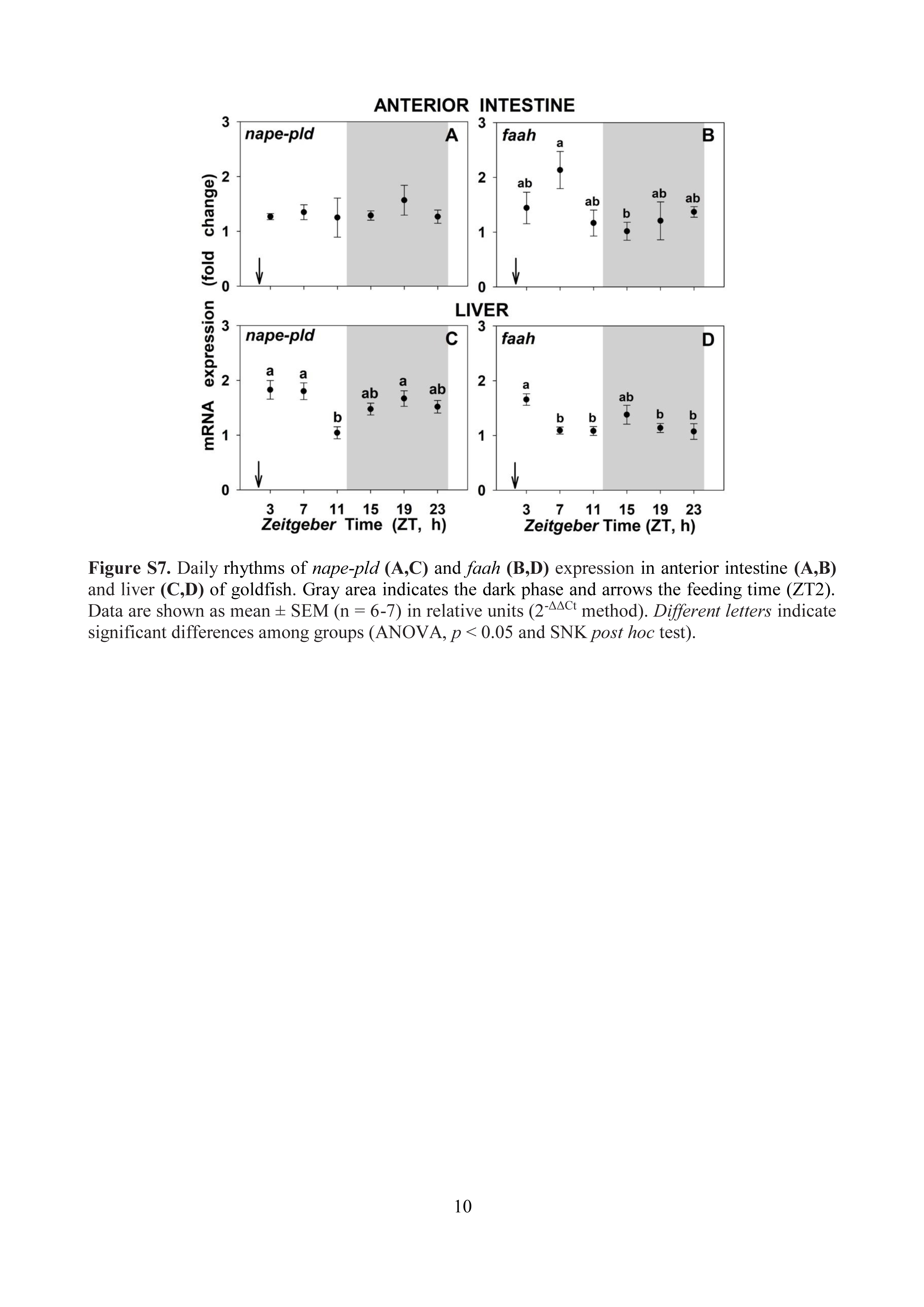

Supplement: Supplementary file 9 [file Image_7.jpg]

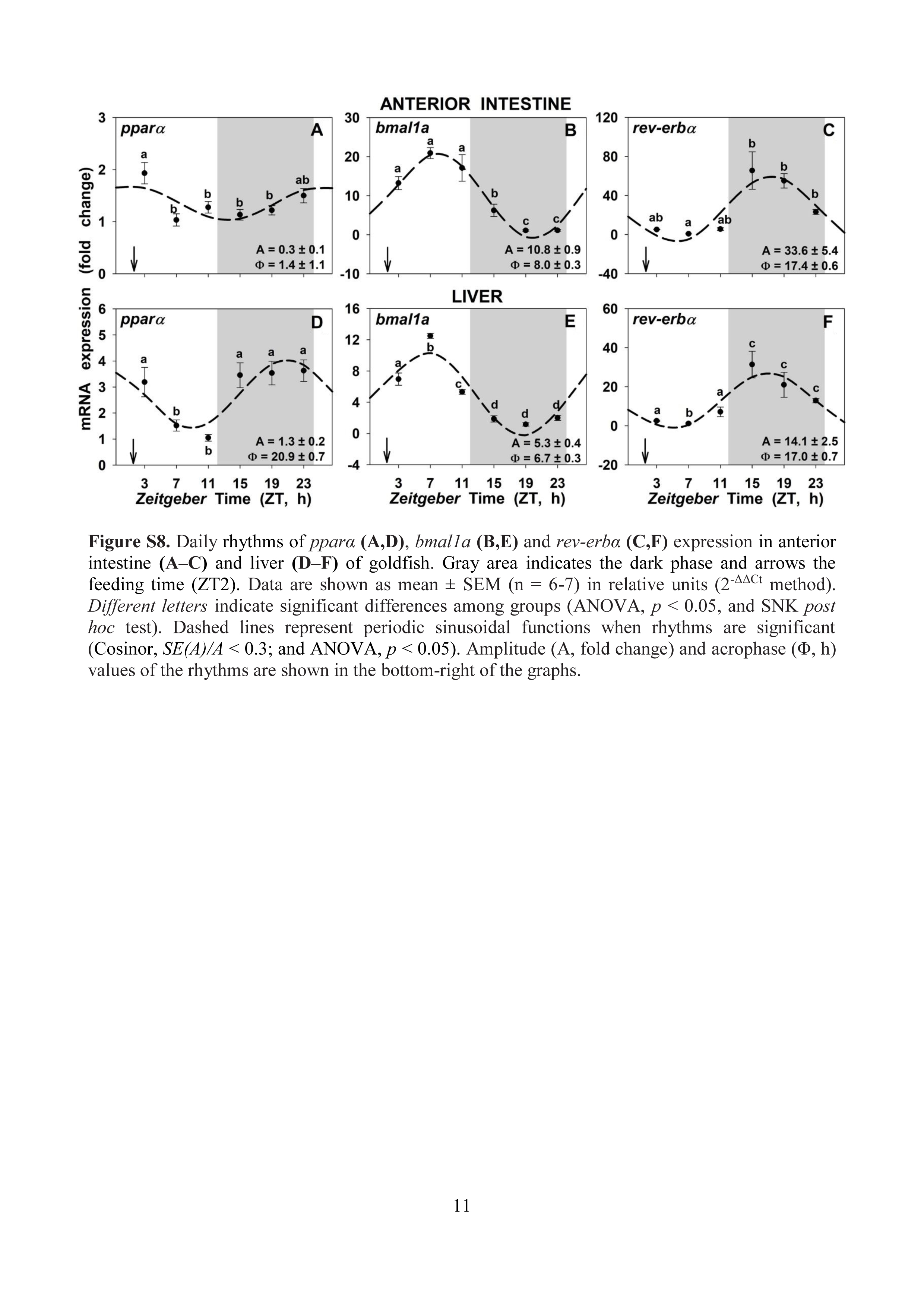

Supplement: Supplementary file 10 [file Image_8.jpg]
